# The effects of licit and illicit recreational drugs on prospective memory: a meta-analytic review

**DOI:** 10.1007/s00213-019-05245-9

**Published:** 2019-05-16

**Authors:** Bradley Platt, Ciarán O’Driscoll, Valerie H. Curran, Peter G. Rendell, Sunjeev K. Kamboj

**Affiliations:** 1 Clinical Psychopharmacology Unit, University College London, London, UK; 2 Institute of Cognitive Neuroscience, University College London, London, UK; 3 Cognition and Emotion Research Centre, Australian Catholic University, Melbourne, Australia

**Keywords:** Prospective memory, Alcohol, Cannabis, Ecstasy, Methadone, Tobacco, Opiate, Methamphetamine

## Abstract

**Rationale:**

There are no recent reports summarising the magnitude of prospective memory (PM) impairments in recreational drug users.

**Objective:**

We performed a meta-analysis of studies (with a parallel group design) examining PM performance in users of common recreational drugs (including alcohol and tobacco) who were not intoxicated during testing. Studies were also evaluated for the presence of methodological bias.

**Methods:**

Twenty-seven studies were included in the meta-analysis following literature searches of MEDLINE, EMBASE and PsycINFO. Effect sizes (standardised mean difference; SMD) were calculated separately for the effects of alcohol, cannabis, ecstasy, methamphetamine and tobacco use. The influences of drug use and study characteristics on effect sizes were explored using meta-regressions. Sources of study bias were also assessed.

**Results:**

Heavy drinkers and regular drug users tended to perform worse than controls on event and time-based PM tasks. Effect sizes (standardised mean differences; SMDs) for event-based PM impairment across the different drug-using groups/heavy drinkers ranged between − 1.10 and − 0.49, with no 95% CI crossing 0.00. SMDs for time-based PM ranged between − 0.98 and − 0.70. Except for the CIs associated with the ES for smokers’ time-based PM performance, no CIs crossed 0.00.

**Conclusions:**

Although all drug-using groups showed moderate-large impairments in event and time-based PM, effect sizes had low precision and moderate-high levels of heterogeneity. In addition, several methodological and reporting issues were identified in the majority of studies. As such, considerable uncertainty remains regarding the role of confounds and the magnitude of PM impairments in non-intoxicated recreational drug users.

**Electronic supplementary material:**

The online version of this article (10.1007/s00213-019-05245-9) contains supplementary material, which is available to authorized users.

## Introduction

The maladaptive use of recreational substances is a major international public health concern. Regular and/or excessive substance use including heavy drinking and smoking is associated with significant levels of mortality, morbidity and social problems, with tobacco and alcohol use being two of the leading risk factors for disability and mortality (World Health Organization 2009). Most psychoactive compounds are neurotoxic, especially after large or repeated doses, and in the case of illicit drugs, often contain adulterants with unknown associated toxicity. As such, impairments in cognitive functioning following acute or chronic drug use (Fernández-Serrano et al. 2011) and consequent interference with daily activities (Grafman and Litvan 1999) is perhaps unsurprising. One domain of special relevance to everyday functioning, for which there is mounting experimental evidence of drug-induced impairments, is prospective memory (PM).

PM refers to the ability to remember to carry out intended actions in the future (Brandimonte et al. 1996). According to McDaniel and Einstein’s (2000) multi-process model, PM relies on either spontaneous retrieval (in which the occurrence of a triggering event promotes retrieval of the intended action from long-term memory) or strategic monitoring (in which the participant actively monitors the environment for triggering cues). When cognitive processes involved in on-going behaviour support the processes involved in PM, the tasks are defined as ‘focal-cue’ tasks and are thought to rely on spontaneous retrieval. Alternatively, when these do not support the processes involved in PM, the relevant tasks are defined as ‘non-focal’ and depend upon strategic monitoring. In addition, a distinction is made between event- and time-based PM tasks with the former relying more on spontaneous, cue-driven retrieval and the latter on strategic monitoring (Einstein et al. 1995). Whilst event-based tasks require participants to perform an intention in response to an external cue (e.g. “buy a birthday present at the shopping centre”), time-based tasks require participants to perform an intention at a specific time or after a delay (e.g. call the plumber at 2 pm).

Severe impairments in PM likely have commensurately severe consequences for daily functioning through a failure to enact intended (adaptive) actions. In those individuals who are attempting to refrain from drug use, PM impairments may also interfere with the ability to apply planned relapse prevention strategies. However, while the negative consequences of psychoactive drug use on PM are commonly reported, methodological limitations in a significant amount of existing research mean that the nature and severity of PM problems that are attributable to specific drugs remains unclear.

Subjective measures of meta-cognition suggest impairments in PM amongst heavy drinkers (Heffernan et al. 2002; Ling et al. 2003, 2010), cannabis users (Montgomery and Fisk 2007; Fisk and Montgomery 2008) and ecstasy users (Heffernan et al. 2001a, b; Rodgers et al. 2001, 2003; Hadjiefthyvoulou et al. 2010). However, the self-report measures used to investigate these metacognitive effects may be susceptible to biases related to lifestyle (Uttl and Kibreab 2011), anxiety (Bedi and Redman 2008a), retrospective memory (Brown and Craik 2000) and social acquiescence (Ling et al. 2010). Furthermore, self-reported complaints about PM have limited predictive validity and are, at best, only weakly correlated with performance on everyday PM-dependent tasks (Hertzog et al. 2000).

In light of these measurement issues, some studies have investigated drug effects on PM with behavioural rather than self-report measures. Similar to the above findings, these studies have shown, for example, that acute intoxication from alcohol (Leitz et al. 2009; Montgomery et al. 2011; Paraskevaides et al. 2010) and ecstasy (Kuypers et al. 2013; Ramaekers et al. 2009; van Wel et al. 2011) adversely affects PM abilities. However, there are likely substantial differences between the pharmacological effects and pharmacokinetic profiles of single doses of pharmaceutical-grade compounds administered in laboratory settings and those used recreationally in naturalistic contexts. Thus, while lab-based acute drug studies enable tight experimental control, they provide little insight into whether PM deficits extend beyond the acutely or sub-acutely intoxicated state.

In this study, we therefore use a meta-analytic approach to review the chronic effects of psychoactive drugs on PM in non-intoxicated users. In addition to determining effect sizes of PM impairment, reviewed studies were also assessed for susceptibility to bias and effects of important confounds such as premorbid intelligence and age (Cherry and LeCompte 1999). We focus on the effects of licit and illicit drugs on event and time-based PM performance.

## Method

### Search methods

Articles were identified through a literature search of MEDLINE (1946 to March 2017), EMBASE (1980 to March 2017) and PsycINFO (1806 to March 2017), using keywords and synonyms of the following common categories of licit and illicit recreational drugs: ‘alcohol’ OR ‘cannabis’ OR ‘tobacco’ OR ‘amphetamine’ OR ‘cocaine’ OR ‘opioid.’ The ‘AND’ operator was used for the keyword: ‘prospective memory’ (see supplementary material for search strategy: S1). ‘Binge drink?’ was included as an additional alcohol-specific search term. Electronic searches were supplemented by hand searches of reference lists, although this yielded no additional studies.

**Fig. 1:**
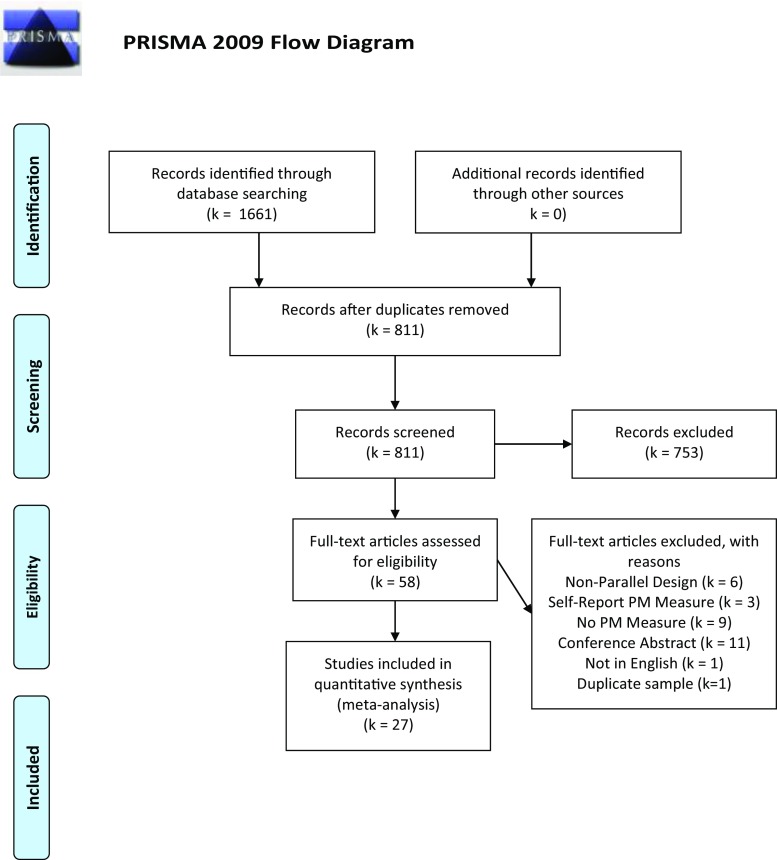
Primsa flowchart

Studies were included if they were full-text journal articles and met the following criteria: (1) were published in an English language peer-reviewed journal, (2) the primary aim was to examine the effects of psychoactive drug-use on PM performance, (3) used a parallel group design with a control condition (consisting of non-using or light and/or infrequent users) and experimental condition (participants who frequently and/or excessively used the primary drug), (4) evaluated PM using a behavioural rather than self-report measure and (5) used a behavioural task that tapped the full complement of cognitive activities required for PM (see below). The process of study selection and exclusion followed PRISMA guidelines (Fig. 1).

### Inclusion criteria for prospective memory tasks

According to influential conceptual models of the cognitive processes involved in successful PM performance, there are four sequential stages in the execution of an intended future action (e.g. Knight 1998). The initial stage involves the formation and encoding of an intention and action plan as well as an evaluation of potential factors that could optimise or impede performance. Secondly, there is a retention interval where other cognitive activities can potentially interfere with the rehearsal of the encoded intention. The third stage involves self-initiated retrieval of the intention, where a target cue triggers the effortful and controlled search for the intention in memory. Finally, actual retrieval and execution of the intention occurs. These features of PM require that valid objective measures of PM incorporate the constituent cognitive processes or activities of these four stages. In particular, PM tasks should incorporate a delay between the encoding and execution of the intention with the delay filled with a secondary on-going task. Furthermore the task must incorporate cues or prompts to initiate intention retrieval without external reminders. For the current review, tasks that did not incorporate all of these features were not considered to be construct-valid assessments of the full range of PM competencies, and studies using such tasks were therefore not included.

### Data extraction

Quantitative data (mean, standard deviation and sample size) on PM task performance were extracted and coded by PM type (event vs. time) by the first and second authors.

Table 1 outlines salient participants and study characteristics extracted from included studies. We also determined whether reliability and validity of PM tasks had been formally established and rated the tasks accordingly (see below). These ratings were used in a moderator analysis. Fifteen distinct tasks were used to assess prospective memory across the included studies (Table 1). These were the Cambridge Prospective Memory Test (CAMPROMPT; Wilson et al. 2005), Video-Based Prospective Memory Task (V-B PMT; Titov and Knight 2001), Memory for Intentions Screening Test (MIST; Raskin et al. 2010), belonging subtest of the Rivermead Behavioural Memory Test (RBMT; Wilson et al. 1985), Six Elements Test (SET; Kliegel et al. 2007), Designated Crosses Test (DC; Bedi and Redman 2008b), Virtual Week (VW; Rendell and Craik 2000), Fruit Test (FT; Cuttler et al. 2012), Pattern Test (Hadjiefthyvoulou et al. 2010), Pattern Recognition Prospective Memory Task (PRPMT; Gallagher et al. 2014), Fatigue Time-Based Prospective Memory Test (FTBPT; Gallagher et al. 2014), Computerised Shopping Task (CDT; Laloyaux et al. 2012), Short-Interval Prospective Memory Task (SIPMT; McHale and Hunt 2008), Jansari-Agnew-Akesson-Murphy Task (JAAMT; Jansari et al. 2014) and Prospective Remembering Video Procedure (PRVP; Seed et al. 2005). Ratings of ‘established psychometric properties’ were based on previously reported psychometric properties of the tasks (see S4) using a 1–3 scale (1 = measures with acceptable test-retest reliability and concurrent validity; 2 = concurrent validity (objective measures) and questionable or no evidence of test-retest reliability or split-half reliability, and 3 = no evidence of reliability and where concurrent validity was based on self-report measures). In four studies, the Virtual Week was used to assess prospective memory which makes a distinction between ‘regular’ and ‘irregular’ PM tasks (Griffiths et al. 2012; Platt et al. 2016; Rendell et al. 2007, 2009). Here, we only extracted participants’ ‘irregular’ PM performance as the task demands are comparable with other measures of PM.

**Table 1:** Demographic and study details by drug group

	Experimental			Control		Prospective memory measure			Drug use
Study	Subgroups	Male:Female	Age mean(SD)	Male:Female	Age mean(SD)	Name	Event or time	Measure quality	Lifetime dosage
Alcohol									
Griffiths et al. (2012)	None	16/8	42.00 (8.74)	16/8	41.90 (8.63)	VW	Event and time	1	3
Heffernan et al. (2010a)	None	7/14	18.70 (0.46)	5/24	18.60 (0.48)	PRVP	Event	2	2
Heffernan and O’Neill (2012)	None	14/14	24.10 (5.30)	12/16	24.30 (5.45)	CAMPROMPT	Event and time	2	2
Marshall et al. (2016)	None	15/25	22.30 (4.10)	6/19	22.55 (4.16)	CAMPROMPT	Event and time	2	2
Laloyaux et al. (2012)	None	17/3	46.0 (10.72)	17/3	45.8 (10.63)	CST	Event	3	3
Platt et al. (2016)	None	11/8	25.55 (2.36)	13/5	27.60 (1.59)	VW	Event and time	1	2
Weinborn et al. (2011)	None	9/12	19.50 (2.10)	11/20	19.70 (1.6)	MIST	Event and time	1	1
Cannabis									
Bartholomew et al. (2010)	None	20/25	Not reported	17/28	Not reported	V-B PMT	Event	2	2
Bedi and Redman (2008b)	None	26/22	21.70 (3.50)	21/19	23.10 (3.70)	DC	Event	3	2
Cuttler et al. (2012)	Experimental/	19/29	20.75 (2.78)	10/38	19.71 (2.59)	FT	Event	2	1
	Chronic users	25/23	20.42 (2.52)						
Gallagher et al. (2014)	None	17/21	21.47 (3.00)	17/48	20.64 (2.23)	PRPMT and FTBPT	Event and time	3	2
Hadjiefthyvoulou et al. (2011b)	None	5/7	21.92 (1.56)	2/16	20.44 (2.28)	CAMPROMPT	Event and time	2	2
McHale and Hunt (2008)	None	10/8	21.60 (1.10)	10/10	21.40 (1.60)	SIPMT and long interval	Event and time	3	?
Montgomery et al. (2012)	None	13/7	21.05 (1.79)	7/13	20.30 (4.65)	JAAMT	Event and time	3	3
Ecstasy/MDMA									
Bedi and Redman (2008a, b)	None	24/21	22.80 (3.0)	21/19	23.10 (3.70)	DC	Event	3	2
Gallagher et al. (2014)	None	51/51	21.85 (2.98)	17/48	20.64 (2.23)	PRPMT and FTBPT	Event and time	3	2
Hadjiefthyvoulou et al. (2011a)	None	14/28	21.67 (3.61)	5/26	21.03 (3.25)	Belonging test of RBMT	Event	1	3
Hadjiefthyvoulou et al. (2011b)	None	17/12	21.17 (1.79)	2/16	20.44 (2.28)	CAMPROMPT	Event and time	2	3
Montgomery et al. (2010)	None	13/10	23.22 (4.56)	9/17	21.92 (2.27)	JAAMT	Event and time	3	2
Rendell et al. (2007)	None	14/13	21.30 (1.96)	15/19	20.60 (1.40)	VW	Event and time	1	?
Weinborn et al. (2011)	None	12/19	21.40 (3.30)	11/20	19.70 (1.60)	MIST	Event and time	1	1
Zakzanis et al. (2003)	None	12/3	24.10 (5.60)	14/3	23.40 (2.00)	Belonging test of RBMT	Event	1	2
Methamphetamine									
Iudicello et al. (2011)	None	36/3	41.60 (8.80)	11/15	40.60(13.80)	MIST	Event and time	1	2
Rendell et al. (2009)	None	12/8	27.50 (5.21)	12/8	28.20 (5.00)	VW	Event and time	1	2
Tobacco									
Behrendt et al. (2015)	None	14/9	25.30 (4.26)	8/12	21.80 (2.63)	SET	Event	3	2
Heffernan et al. (2010b)	None	5/13	25.20 (5.28)	3/19	22.50 (4.21)	CAMPROMPT	Event and time	2	2
Heffernan et al. (2012)	Smoker	11/16	22.40 (5.13)	3/21	19.00 (2.22)	RWPMT	Event		
	Previous Smoker	3/15	23.70 (5.99)					3	2
Heffernan et al. (2013)	Second-Hand	13/14	22.00 (1.46)	10/18	22.80 (6.03)	CAMPROMPT	Event and time		
	Current Smoker	12/12	24.4 (4.79)						
Heffernan et al. (2013)	None	14/25	21.10 (2.63)	18/21	20.60 (2.10)	V-B PMT	Event	2	2
Heffernan et al. (2014a)	None	18/6	21.20 (2.04)	14/10	20.50 (0.97)	CAMPROMPT	Event and time	2	2
Jansari et al. 2013	None	15/21	27.73 (8.27)	18/18	28.94 (11.50)	JAAMT	Event	3	2
McHale and Hunt (2008)	None	10/10	21.20 (1.28)	10/10	21.40 (1.60)	SIPMT	Event	3	?
Marshall et al. (2016)	None	6/14	27.15 (6.80)	6/19	No report	CAMPROMPT	Event and time	2	2
Opiates									
Terrett et al. (2014)		18/8	38.31 (7.46)	16/14	39.47 (7.94)	VW	Event and time	1	1

Notes: Cambridge Prospective Memory Test (CAMPROMPT; Wilson et al. 2005), Video-Based Prospective Memory Task (V-B PMT; Titov and Knight 2001), Memory for Intentions Screening Test (MIST; Raskin et al. 2010), belonging subtest of the Rivermead Behavioural Memory Test (RBMT; Wilson et al. 1985), Six Elements Test (SET; Kliegel et al. 2007), Designated Crosses Test (DC; Bedi and Redman 2008b), Virtual Week (VW; Rendell and Craik 2000), Fruit Test (FT; Cuttler et al. 2012), Pattern Test (Hadjiefthyvoulou et al. 2010), Pattern Recognition Prospective Memory Task (PRPMT; Gallagher et al. 2014), Fatigue Time-Based Prospective Memory Test (FTBPT; Gallagher et al. 2014), Computerised Shopping Task (CDT; Laloyaux et al. 2012), Short-Interval Prospective Memory Task (SIPMT; McHale and Hunt 2008), Jansari-Agnew-Akesson-Murphy Task (JAAMT; Jansari et al. 2004), and Prospective Remembering Video Procedure (PRVP; Seed et al. 2005)

In addition, estimated lifetime drug/alcohol dose was determined from available information in each study. Within subgroup, *z*-scores were calculated. Participant samples were then rated as 1 = low (< − 1SD), 2 = moderate (− 1 to 1 SD), and 3 = high (> 1 SD) lifetime exposure to a specific drug. There was no relevant data to estimate lifetime dose in two studies (McHale and Hunt 2008; Rendell et al. 2007).

### Study quality rating

We also rated studies on robustness of design and susceptibility to bias (rating was conducted independently by the first two authors; disagreements in ratings were resolved through discussion). Bias in participant selection, case definition (i.e. allocation to groups based on objective/standardised measures of drug-use) and blinding of participants and investigators was assessed (Downs and Black 1998; Sanderson et al. 2007; Wells et al. 2011). Additional items on methodological quality on domains considered by the authors to be especially relevant for drug studies of PM were also included in the quality rating scheme (matching of groups on age, intelligence, drug-use and verification of non-intoxication). All domains of bias were assigned a high, low or unclear risk of bias. If information was not available in the publication, quality items were coded as ‘not available.’ Details on criteria for quality ratings and a summary of the authors’ judgements about each risk of bias item for each included study are included in the supplementary section (S2 and S3).

### Analyses

Effect sizes based on the standardised mean difference (SMD) between control and drug-using groups were calculated using random effects models. Larger negative effect sizes indicated that the mean performance of the substance-using group was lower than the control group. Effect sizes in the range 0.20–0.49 were defined as small, 0.50–0.79 as moderate and ≥ 0.80 as large (Cohen 1988).

Analyses were conducted using the Cochrane Collaboration’s Review Manager software (RevMan version 5.3), and in *R* using the metafor package (Viechtbauer 2010). Random effects models (maximum likelihood estimator) were used, with the assumption that studies had different true effect sizes, as such the combined effect size represents a distribution of effect sizes (Higgins and Green 2011). Heterogeneity was assessed using a point estimate of the amongst-study variance of true effects (*τ*^2^) and the approximate proportion of total variability (*I*^2^), where an *I*^2^ of 25%, 50% and 75% is considered small, moderate and large, respectively (Higgins 2008). When moderate or high heterogeneity was observed, interpretation emphasised the range of likely combined effect sizes (confidence intervals) rather than a single-summary effect size. Sensitivity analysis involved stepwise removal of studies to assess the impact of their removal on high levels of heterogeneity.

Random effects meta-regressions (maximum likelihood estimator) were conducted to relate the effect sizes to characteristics of the studies (lifetime dosage and measures used). This form of analysis can help to explain the heterogeneity between studies providing estimates of the difference in effect between studies rated on the three levels of each categorical moderator variable. Due to the number of studies, it was not possible to conduct analyses on specific drug groups.

## Results

**Fig. 2:**
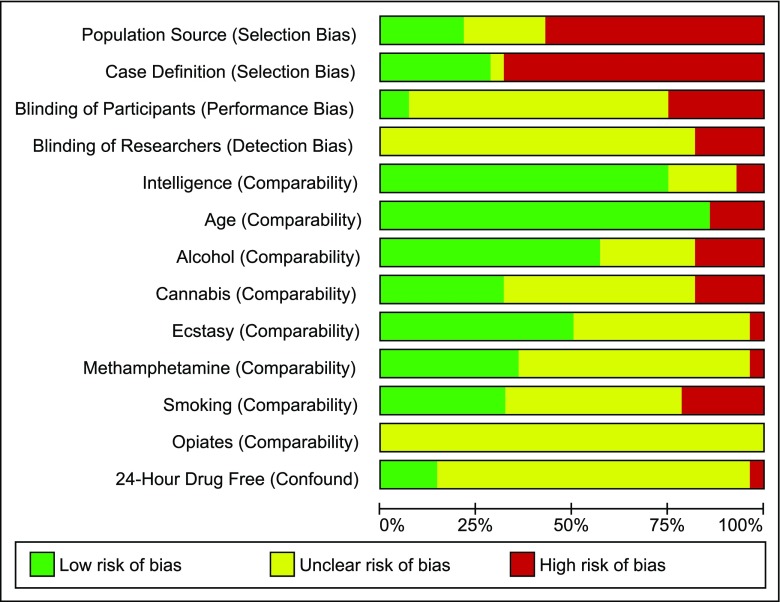
Risk of bias summary: Prevalence of bias for each domain presented as percentage of studies with high, low or unclear risk of bias

Twenty-seven studies fulfilled inclusion criteria and provided data for meta-analyses. Figure 2 summarises risk of bias ratings for all included studies. Certain methodological features were consistently rated as having a high risk of bias (e.g. population sourcing; case definition). For other features (e.g. blinding of experimenters and participants), reporting was poor (unclear bias). Alternatively, a number of study features were less commonly associated with bias across the studies as a whole (comparability of control and drug-using groups in terms of participant age, intelligence and alcohol use).

**Fig. 3:**
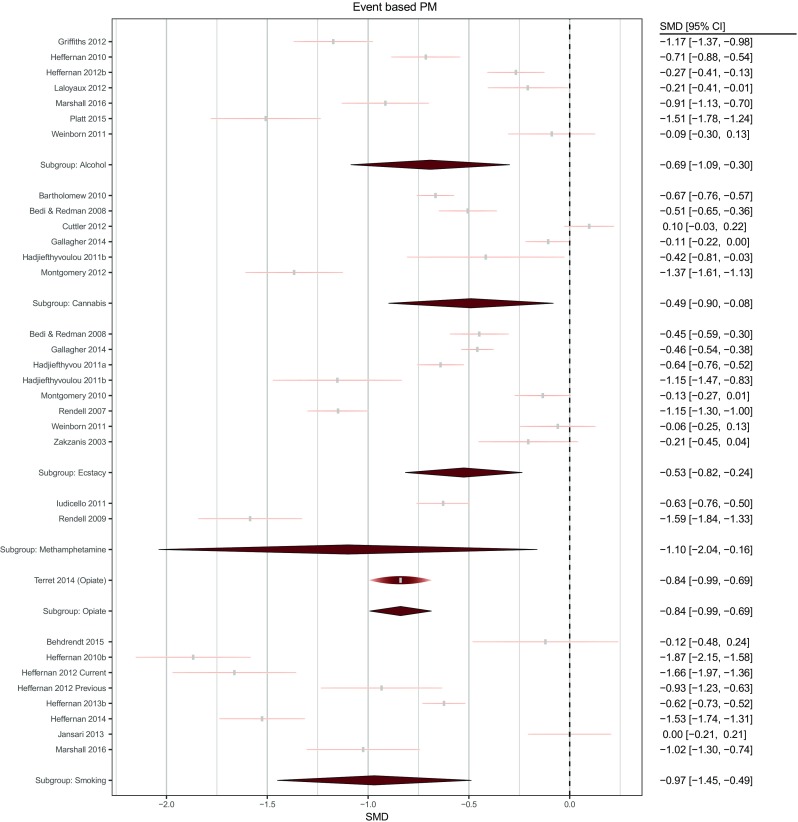
Forest plot of comparisons for event-based PM tasks across drug conditions. The width of each node is identical to the width of the confidence interval. The height of each raindrop is scaled with respect to its relative meta-analytic weight considering all studies within the subgroup

**Fig. 4:**
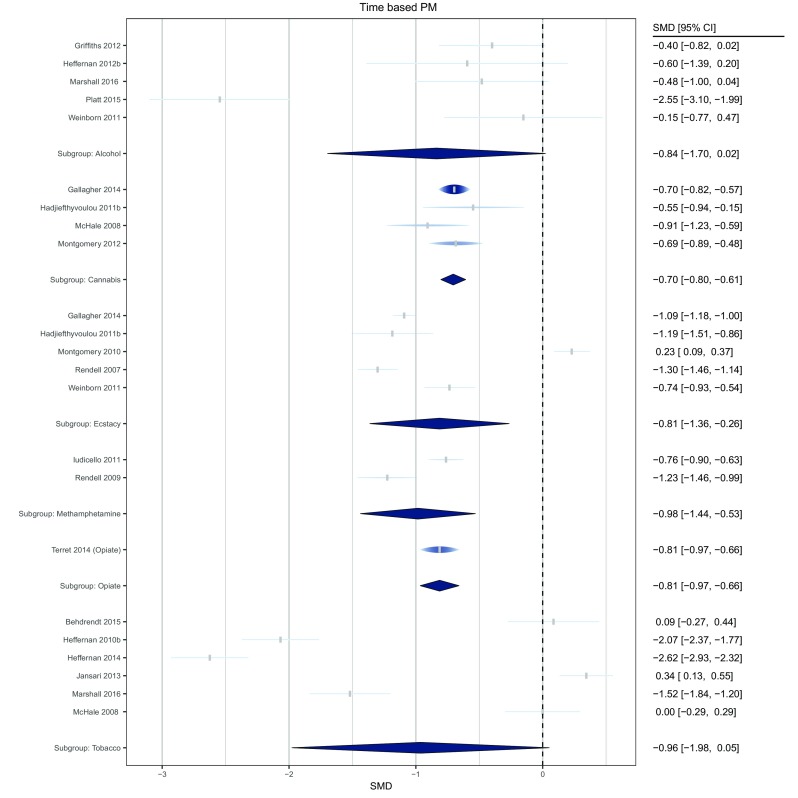
Forest plot of comparisons for time-based PM tasks across drug conditions. The width of each node is identical to the width of the confidence interval. The height of each raindrop is scaled with respect to its relative meta-analytic weight considering all studies within the subgroup

Forest plots for event and time-based PM comparisons are displayed in Figs. 3 and 4. Means, standard deviations and sample sizes used in effect size calculations are included in the supplementary material (S5 and S6).

### Alcohol

Seven studies compared heavy drinkers (total *n* = 172), with an estimated total lifetime use ranging between 800 and 20,000 units, with control groups (total *n* = 148) on event-based PM performance. As indicated in Fig. 3, alcohol groups performed worse than control groups and the true effect size lays in the small to large range. There was a moderate degree of heterogeneity (*τ*^2^ = 0.164, *I*^*2*^ = 62%) although no individual study’s removal appreciably reduced heterogeneity.

Of the seven alcohol studies, five also compared heavy drinkers (total *n* = 131) with control groups (total *n* = 99) on time-based PM (Fig. 4). Again, alcohol groups performed worse than controls, with the true effect ranging from approximately zero to large. There was a high level of heterogeneity (*τ*^2^ = 0.7, *I*^*2*^ = 87%). However, a single study (Platt et al. 2016) appeared to disproportionately contribute to heterogeneity. Its removal not only eliminated heterogeneity (*τ*^2^ = 0, *I*^*2*^ = 0%), but also reduced the overall effect size from SMD = − 0.84 (95% CI; − 1.70 to − 0.02) to − 0.43 (95% CI; − 0.72 to − 0.13).

### Cannabis

Six studies compared cannabis users (total *n* = 205) with an estimated total lifetime use ranging between 312 and 10,699 joints, with healthy controls (total *n* = 151), on event-based PM tasks, with cannabis groups performing worse, and the likely true effect size between negligible and large. Accordingly, there was high heterogeneity (*τ*^2^ = 0.159, *I*^2^ = 68%). Excluding Montgomery et al. (2012) not only reduced heterogeneity (*τ*^2^ = 0.053, *I*^*2*^ = 43%) but also reduced the effect size from SMD = − 0.49 (95% CI; − 0.90 to − 0.08) to SMD = − 0.31 (95% CI; − 0.63 to − 0.004).

Four studies compared a cannabis-using group (total *n* = 88) with healthy controls (total *n* = 71), on a time-based PM task. There was a moderate combined effect size, with the cannabis groups performing worse, and no heterogeneity (*τ*^2^ = 0, *I*^*2*^ = 0%).

### Ecstasy

Eight studies compared an ecstasy group (*n* = 272) with varying histories of quantities of ecstasy consumed (estimated total lifetime use ranging between 30 and 668 tablets) with healthy controls (*n* = 196), on an event-based PM task, with ecstasy groups performing worse, displaying a small to large effect. There was moderate heterogeneity (*τ*^2^ = 0.065, *I*^*2*^ = 45%).

Five studies compared an ecstasy group (total *n* = 213) with healthy controls (total *n* = 127), on a time-based PM task, with ecstasy groups performing worse, which also indicated a small to large effect. There was high heterogeneity (*τ*^2^ = 0.32, *I*^*2*^ = 80%). Excluding Montgomery et al. (2010) explained the heterogeneity (*τ*^2^ = 0, *I*^*2*^ = 0%) and increased the effect size from SMD = − 0.81 (95% CI; − 1.36 to − 0.26) to SMD = − 1.08 (95% CI; − 1.36 to − 0.809).

### Methamphetamine

Two studies compared a methamphetamine group with an estimated total lifetime use ranging between 720 and 1058 g, (total *n* = 59) with healthy controls (total *n* = 46), on an event-based PM task, with methamphetamine groups performing worse, indicating a small to large effect. There was high heterogeneity (*τ*^2^ = 0.359, *I*^*2*^ = 78%).

The same two studies compared groups on a time-based PM task, with methamphetamine groups performing worse, indicating a moderate to large effect size with low heterogeneity (*τ*^2^ = 0.013, *I*^*2*^ = 12%).

### Opiates

One study compared an opiate group (long-term heroin users enrolled in opioid replacement treatment estimated lifetime use of 4272 g; *n* = 26) with controls (*n* = 30) on an event- and time-based task, both indicating a moderate to large effect, with the opiate group performing worse than healthy controls.

### Smoked tobacco/nicotine

Seven studies (eight group comparisons) compared a tobacco-smoking group (total *n* = 175), with an estimated total lifetime ranging between 10,610 and 117,511 cigarettes, with healthy controls (total *n* = 153), on an event-based PM task. These indicated that tobacco groups tended to perform worse than controls, with a moderate to large combined effect and moderate heterogeneity (*τ*^2^ = 0.344, *I*^*2*^ = 74%). No study appeared to explain heterogeneity.

Six studies compared a tobacco group (total *n* = 111) with healthy controls (total *n* = 100), on a time-based PM task, indicating a negligible-large effect. There was no significant difference between groups with studies that reported contrasting findings. There was high heterogeneity (*τ*^2^ = 1.46, *I*^*2*^ = 91%). Again, no individual study’s removal reduced heterogeneity.

### Meta-regression

Meta-regression analyses revealed an influence of PM task’s reliability/validity rating (*Q*_model_ (2) = 6.86, *p* = 0.032, *R*^2^ = 22%). Studies using measures with acceptable test-retest reliability and concurrent validity displayed a large effect size SMD (*k* = 8) = − 0.953 95% CI (− 1.404, − 0.502, *p* < 0.001). Studies reporting concurrent validity (with objective measures) and questionable or no evidence of test-retest reliability or split-half reliability displayed a larger overall effect size with wider CIs SMD (*k* = 7) = 1.274 (0.657 to 1.890, *p* < 0.001). While tasks with no evidence of psychometric properties (reliability and validity) displayed smaller effect sizes SMD (*k* = 8) = − 0.357 95% CI (− 0.77, − 0.043, *p* = 0.08). However, this was not observed for the event-based PM (*Q*_model_ (2) = 1.959, *p* = 0.367, *R*^2^ = 0%). Categorisation of lifetime dosage displayed no explanatory value.

## Discussion

This study used a meta-analytic approach to examine the magnitude of PM impairments across studies of various licit and illicit recreational drugs. Our analyses revealed that regular users of alcohol, cannabis, ecstasy and opiates performed significantly worse than controls on event- and on time-based PM tasks. Regular smokers performed significantly worse than controls on event-based but not time-based PM tasks, although the effect size estimate for the latter was particularly imprecise with the true effect lying between very large (SMD approximately − 2) and negligible. The effect sizes for impairments on event-based PM tasks were moderate–large in smokers and heroin users (the latter based on a single study), small–large for alcohol, ecstasy and methamphetamine use, and negligible–large for cannabis. Effect sizes for time-based PM tasks were moderate–large for methamphetamine and opiate use, moderate for cannabis, small–large for ecstasy and negligible–large for tobacco and alcohol. Heterogeneity influenced a number of effect size estimates. As an illustration of the effects of individual studies in determining heterogeneity (as well as overall effect size), exclusion of the study by Platt et al. (2016), resulted in a marked reduction in the heterogeneity of effect sizes in time-based PM performance amongst studies of heavy drinkers, as well as nearly halving the overall effect size.

Despite reflecting the effects of chronic/sub-acute substance use, rather than acute intoxication, the effects summarised here are broadly in line with laboratory studies of acute effects of alcohol on PM (Leitz et al. 2009; Montgomery et al. 2011; Paraskevaides et al. 2010). However, it is important to note that effects of acute alcohol have to date only been investigated in light/social drinkers. The acute-on-chronic effects of alcohol in heavy drinkers have yet to be investigated. In addition, effects reported here on ecstasy (unverified MDMA content) are similar to those found following acute MDMA administration in laboratory settings (Kuypers et al. 2013; Ramaekers et al. 2009; van Wel et al. 2011). In contrast to the effects of smoked tobacco/nicotine reported here, laboratory-based studies on the acute effects of nicotine have found nicotine to improve PM performance of smokers (Dawkins et al. 2013; Jansari et al. 2013; Rusted et al. 2005; Rusted and Trawley 2006) and ‘non-smokers’ (Rusted and Trawley 2006), with the exception of one study showing no effect of nicotine on PM in never-smokers (Jansari et al. 2013).

A number of methodological issues were identified within the reviewed studies. For example, there was a high level of polydrug use across studies, particularly in the ecstasy studies. This highlights the difficulty of distinguishing discrete drug effects in studies of non-acutely intoxicated users. Specifically, it is unclear to what extent the observed impairments were due to the primary drug of interest versus other regularly used drugs, or indeed the combination of drugs. In studies of smokers for instance, participants had high levels of alcohol use, some reaching levels as high as those reported in the studies of heavy drinkers. The lack of comprehensive reporting on general drug use in many studies makes it difficult to ascertain the true effect of polydrug use on the effects reported here. In addition, since assessment of bias relating to drug use in the previous 24 h was ‘unclear’ in most studies, the role of abstention (or withdrawal) or acute drug effects in the impairments reported here also remains unclear. Related confounds also complicate interpretation. For example, Ludicello et al. (2011) reported high prevalence of AIDS in their sample of methamphetamine users (60%) introducing two potential confounds; the cognitive impairment of HIV/AIDS (Watkins and Treisman 2015) and the possible effects of antiretroviral therapy on cognitive performance (Liner et al. 2008).

Many studies had recruited participants from student populations. Higher levels of education and/or cognitive ability (intelligence) in the drug-using groups may have protected against the cognitive impairing effects of drug use (in line with a cognitive reserve hypothesis) and contribute to some of the smaller effect sizes found in the meta-analyses. Other studies recruited their drug-using groups from outside student subject pools and in clinical settings or detox treatment (e.g. Bedi and Redman 2008b; Griffiths et al. 2012; Terrett et al. 2014; Weinborn et al. 2011). For example, Griffiths and colleagues recruited their experimental group from a residential substance misuse service for those with alcohol dependence, and their control group from a university subject pool consisting of students and members of the public. These studies might have greater clinical relevance but they also have a potential for greater bias, associated with for example, the presence of comorbid psychiatric disorders (Hasin et al. 2007) and lower socioeconomic status (Hackman et al. 2010) in more severely affected drug users found in clinical settings. For example, schizophrenia is associated with greater PM impairments than affective disorders (Burton et al. 2018). By recruiting comparison groups from the same or similar populations, matching groups on relevant demographics (e.g. age, educational achievement, premorbid IQ) and using conservative inclusion criteria, future research should aim to increase the internal and external validity of studies.

Surprisingly, lifetime drug dose did not appear to moderate the size of PM impairments. This may have been a result of the relatively crude categorical rating scheme used here, as lifetime dose is a continuous variable. However, this was chosen given the variance in reporting methods on quantifiable drug use. An alternative categorical rating scheme might have been more informative, examining the moderating influence of regularity of drug use (e.g. daily versus less regular use). Moderation analyses within each drug group might have been more informative in determining the moderating role of lifetime drug dose, since cumulative neurotoxicity may be more severe for some drugs (e.g. alcohol and methamphetamine) than others (e.g. smoked tobacco). However, given the small number of studies per drug grouping, such an analysis would have been underpowered.

In most studies, participants were allocated to groups with un-validated measures of drug use. The exception was Weinborn et al. (2011) and Platt et al. (2016). In these studies, a cut-off score on a validated measure of alcohol use was used for group allocation. However, no further verification of drug/alcohol use was used and as such, healthy controls may have under-reported the quantity and frequency of drug/alcohol use (e.g. see Townshend and Duka 2002). Such under-reporting might have reduced the differences in mean PM performance between control and drug-using groups.

There are a number of limitations inherent in the present review. First, as already noted, the number of studies per drug class was relatively small, which may have adversely affected the precision of effect size estimates. Second, different measures of PM were used across the different studies, with some measures having limited evidence of adequate psychometric properties. Thirdly and relatedly, the effect sizes were characterised by moderate to high levels of heterogeneity, limiting the strength of conclusions about true effect sizes. Fourthly, we limited the inclusion of studies to those covering relatively common drugs associated with problematic use. Other major drug classes, including various novel psychoactive substances that are known to have cognitive impairing effects, were not examined.

## Summary

In studies of non-acutely intoxicated recreational drug users, PM impairments were found in all drug-using groups (heavy drinkers, smokers, and cannabis, ecstasy, methamphetamine or opiate users). However, there were no prospective studies on drug use and PM deficits with a parallel group design, and as such it remains unclear whether PM deficits are a risk factor for or consequence of recreational drug use. A number of methodological limitations/sources of bias were identified in most studies. Furthermore, there were high levels of methodological and effect size heterogeneity across studies which limit the strength of conclusions that can be drawn about the true effect size of PM impairments for different drugs. Future studies should seek to address the identified methodological issues and also investigate the effects of different patterns of recreational drug use on event and time PM tasks of differing demand levels.

## Electronic supplementary material

ESM 1 (DOC 188 kb)
