# Supplementary material for: The effects of licit and illicit recreational drugs on prospective memory: a meta-analytic review
Source: Psychopharmacology (Berl). 2019 May 16;236(4):1131–43. doi: 10.1007/s00213-019-05245-9 (PMC6591206; doi:10.1007/s00213-019-05245-9)
Supplement: Supplementary file 1 — (DOC 188 kb) [file 213_2019_5245_MOESM1_ESM.doc]

**S1: Search strategy**

1. (alcohol$ or drink$ or ethanol).ti,ab.
2. (ayahuasca or banisteriopsis or dimethyltryptamine or dmt or psychotria).ti,ab.
3. ((bath adj salt$) or cathinone$).ti,ab.
4. cocaine,ti,ab.
5. (gammahydroxybut$ or gamma-hydroxybut$ or y-hydroxybut$ or 4-hydroxybut$ or GHB)ti,ab.
6. ketamine.ti,ab.
7. (khat or cathinone$ or benzoylethanamine$ or b-keto-amphetamine$ or cathine$ or d-norpseudoephedrine$ or (Catha adj edulis)).ti,ab.
8. ((lysergic adj acid) or (LSD or lysergide$)).ti,ab.
9. (mari#uana or cannabi$ or (tetrahydrocannabi$ or tetra-hydrocannabi$ or delta-9-tetrahydrocannabi$ or delta-9-tetra-hydrocannabi$ or THC or CBD)).ti,ab.
10. (amphetamine$ or methylenedioxymethamphetamine$ or MDMA or ecstasy or methamphetamine$ or 4-methylenedioxy$ or 3-methylenedioxy$).ti,ab.
11. (mescaline or peyote or 5-trimethoxyphenethylamine or 4-trimethoxyphenethylamine or 3-trimethoxyphenethylamine).ti,ab.
12. (dextromethorphan or DMX).ti,ab.
13. (phencyclidine or PCP).ti,ab.
14. (codeine or fentanyl or hydrocodone or dihydrocodeinone or hydromorphone or meperidine or methadone or morphine or oxycodone or oxymorphone).ti,ab.
15. (barbiturate$ or pentobarbital or phenobarbital).ti,ab.
16. (benzodiazepine$ or alprazolam or chlorodiazepoxide or diazepam or lorazepam or triazolam).ti,ab.
17. (opium$ or opiod$ or opiate$ or heroin$).ti,ab.
18. (eszopiclone or zalepion or zolpidem).ti,ab.
19. methylphenidate.ti,ab.
20. (mushroom$ or psilocyb$).ti,ab.
21. salvia.ti,ab.
22. (nandrolone or oxandrolone or oxymetholone or stanozolol or cypionate).ti,ab.
23. (smok$ or tobacco or nicotine).ti,ab.
24. ((prospective or future or delay$ or episodic) adj (memor$ or inten$ or foresight or think$)).ti,ab.

**S2. Bias Criteria for Quality Ratings**

|  |  | Low risk of bias indicated by: |
| --- | --- | --- |
| **Selection Bias** | *Population* | Comparison groups recruited from the same population (e.g. hospital, university). Or, inferential statistics demonstrate no difference between comparison groups’ ratio of participants from different recruitment sources. |
| **Allocation bias** | *Case Definition* | Comparison groups allocated by objective and/or standardised measure (e.g. secure records, formal diagnostic interview). With regards to self-report or screening measures, there must be evidence from a peer-reviewed journal on the measure’s predictive validity of alcohol and/or drug use. |
| **Performance Bias** | *Blinding* | Participants could not foresee assignment to comparison groups before or during testing. |
| **Detection Bias** | *Blinding* | Blinding of investigators to participants’ group assignment when assessing subjective-based outcomes. |
| **Comparability** | *Intelligence* | Inferential statistics demonstrate no difference between comparison groups’ performance on tests of premorbid intelligence, time in or level of education, with greater priority for premorbid intelligence measures. |
|  | *Age* | Inferential statistics demonstrate no difference between comparison groups’ age. |
|  | *Alcohol* | Inferential statistics demonstrate no difference between comparison groups’ frequency (life-time and/or current) of alcohol use. |
|  | *Drugs* | Inferential statistics demonstrate no difference between comparison groups’ frequency (life-time and/or current) of drug use. Or, participants’ drug use is ascertained by inclusion criteria for groups. |
|  | *24-Hour* | All participants were free of alcohol or primary drug of interest at the time of testing, as evidenced by a biological measure (e.g. breathalyser, urinalysis or immunoassay). |

**S3. Quality Ratings for studies’ bias**

|  | Population Source | Case Definition | Blinding of Participants | Blinding of Researchers | Intelligence | Age | Alcohol | Cannabis | Ecstasy | Amphetamine | Opiates | Smoking | 24-Hour Testing |
| --- | --- | --- | --- | --- | --- | --- | --- | --- | --- | --- | --- | --- | --- |
| **Alcohol** |  |  |  |  |  |  |  |  |  |  |  |  |  |
| Griffiths et al 2012 | + | - | ? | + | - | - | NA | ? | - | - | ? | + | ? |
| Heffernan et al 2010 | + | + | ? | ? | ? | - | NA | ? | ? | ? | ? | ? | ? |
| Heffernan et al 2012b | ? | + | ? | ? | - | - | NA | - | - | ? | ? | - | ? |
| Laloyaux et al 2012 | - | - | ? | - | - | - | NA | ? | ? | ? | ? | ? | ? |
| Marshall 2016 | - | + | + | + | ? | ? | NA | ? | ? | ? | ? | - | ? |
| Platt et al 2015 | - | - | + | + | - | + | NA | + | + | ? | ? | + | - |
| Weinborn 2011 | + | + | ? | ? | - | - | NA | ? | - | ? | ? | ? | ? |
| **Cannabis** |  |  |  |  |  |  |  |  |  |  |  |  |  |
| Bartholomew 2010 | + | + | ? | ? | ? | - | + | NA | - | - | ? | + | ? |
| Bedi & Redman 2008 | + | - | ? | ? | - | - | - | NA | ? | + | ? | + | - |
| Cuttler et al 2012 | + | + | + | ? | + | - | ? | NA | ? | ? | ? | ? | ? |
| Gallagher et al 2014 | ? | + | ? | ? | - | - | + | NA | ? | ? | ? | - | ? |
| Hadjiefthyvoulou et al 2011b | + | + | + | ? | ? | ? | ? | NA | ? | ? | ? | + | ? |
| McHale & Hunt 2008 | + | + | ? | ? | ? | - | - | NA | - | - | ? | ? | ? |
| Montgomery et al 2012 | + | + | ? | ? | - | - | - | NA | - | - | ? | ? | ? |
| **Ecstasy** |  |  |  |  |  |  |  |  |  |  |  |  |  |
| Bedi & Redman 2008 | + | - | ? | ? | - | - | - | + | NA | + | ? | + | - |
| Gallagher et al 2014 | ? | + | ? | ? | - | + | + | + | NA | ? | ? | + | ? |
| Hadjiefthyvoulou et al 2011a | + | + | ? | ? | - | - | + | + | NA | ? | ? | - | ? |
| Hadjiefthyvoulou et al 2011b | + | + | + | ? | ? | ? | ? | ? | NA | ? | ? | + | ? |
| Montgomery et al 2010 | ? | + | ? | ? | + | - | + | ? | NA | ? | ? | ? | ? |
| Rendell et al 2007 | + | + | ? | ? | - | - | ? | + | NA | ? | ? | ? | ? |
| Weinborn 2011 | + | + | ? | ? | - | - | + | ? | NA | ? | ? | ? | ? |
| Zakzanis et al 2003 | - | - | + | ? | - | - | - | ? | NA | ? | ? | ? | - |
| **Opiate** |  |  |  |  |  |  |  |  |  |  |  |  |  |
| Terret et al 2014 | - | - | ? | ? | + | + | ? | ? | ? | ? | ? | ? | - |
| **Methamphetamine** |  |  |  |  |  |  |  |  |  |  |  |  |  |
| Iudicello 2011 | + | - | ? | ? | - | - | - | + | ? | NA | ? | ? | - |
| Rendell et al 2009 | ? | - | - | ? | - | - | ? | ? | ? | NA | ? | ? | ? |
| **Smoking** |  |  |  |  |  |  |  |  |  |  |  |  |  |
| Behrendt 2015 | + | - | ? | ? | - | + | + | ? | ? | ? | ? | NA | ? |
| Heffernan et al 2010b | - | ? | + | + | - | - | - | ? | ? | ? | ? | NA | + |
| Heffernan et al 2012 | ? | + | ? | ? | - | + | ? | ? | ? | ? | ? | NA | ? |
| Heffernan et al 2013 | ? | - | ? | ? | - | - | - | - | - | - | ? | NA | ? |
| Heffernan et al 2013b | - | + | ? | ? | ? | - | - | - | - | - | ? | NA | ? |
| Heffernan et al 2014 | + | + | + | ? | - | - | - | - | - | - | ? | NA | ? |
| Jansari et al 2013 | + | + | ? | ? | - | - | ? | ? | ? | ? | ? | NA | ? |
| Marshall 2016 | - | + | + | + | ? | ? | ? | ? | ? | ? | ? | NA | ? |
| McHale & Hunt 2008 | + | + | ? | ? | ? | - | - | - | - | - | ? | NA | ? |

*S3****:*** *Risk of bias: review authors' judgements about each risk of bias item for each included study, + = high risk, - =low risk, ? = unclear, NA = not applicable.*

***S4: Rating of psychometric quality of measures of prospective memory***

Measures rated as 1:

Virtual Week (reliability: .84 to .94 for the regular, irregular and time-check tasks) Rivermead  Behavioural   Memory   Test   (reliability: 0.89)

Memory  for  Intentions  Screening  Test  (reliability: 0.78 )

Measures rated as 2:

Camprompt (reliability: 0.64)

Prospective remembering video procedure (Split half reliability .76 and .71)

Measures rated as 3

Jansari-Agnew-Akesson-Murphy  Task

Modified Designated Cross (Bedi & Redman, 2008; Hannon et al., 1995)

Prospective Memory Pattern Recognition Test. (Fisk and Warr 1996)

Fatigue based PM task (Fisk and Warr 1996)

short term time based task (McHale & Hunt, 2008)

modified six elements

Real World PM task

***S5: Event based PM, means, standard deviations, and sample sizes for each study.***

|  |  | Drug Group | |  | Controls | |  |
| --- | --- | --- | --- | --- | --- | --- | --- |
| Study | Drug | Mean | SD | n | Mean | SD | n |
| Griffiths et al., 2012 | Alcohol | 0.37 | 0.33 | 23 | 0.74 | 0.29 | 24 |
| Heffernan et al., 2010 | Alcohol | 10.1 | 2.83 | 21 | 12 | 2.46 | 29 |
| Heffernan et al., 2012b | Alcohol | 14.5 | 1.77 | 28 | 15 | 1.92 | 28 |
| Laloyaux et al., 2012 | Alcohol | 7.35 | 1.59 | 20 | 7.6 | 0.5 | 20 |
| Marshall et al., 2016 | Alcohol | 14.27 | 2.35 | 40 | 16.48 | 2.48 | 13 |
| Platt et al., 2015 | Alcohol | 0.83 | 0.05 | 19 | 0.9 | 0.04 | 18 |
| Weinborn et al., 2011 | Alcohol | 7.19 | 1.12 | 21 | 7.29 | 1.07 | 16 |
| Bartholomew et al., 2010 | Cannabis | 8.8 | 3.09 | 45 | 10.78 | 2.8 | 45 |
| Bedi & Redman et al., 2008 | Cannabis | 25.4 | 4.8 | 48 | 27.6 | 2.7 | 20 |
| Cuttler et al., 2012 | Cannabis | 1.23 | 1.55 | 48 | 1.08 | 1.5 | 24 |
| Gallagher et al., 2014 | Cannabis | -0.74 | 1.11 | 38 | -0.6 | 1.48 | 33 |
| Hadjiefthyvoulou et al., 2011b | Cannabis | 15.08 | 2.39 | 12 | 16 | 1.68 | 9 |
| Montgomery et al., 2012 | Cannabis | 50 | 32.44 | 20 | 88.75 | 22.17 | 20 |
| Bedi & Redman et al., 2008 | Ecstacy | 25.8 | 4.4 | 45 | 27.6 | 2.7 | 20 |
| Gallagher et al., 2014 | Ecstacy | -1.75 | 2.74 | 102 | -0.6 | 1.48 | 33 |
| Hadjiefthyvou et al., 2011a | Ecstacy | 1.19 | 0.77 | 42 | 1.65 | 0.62 | 31 |
| Hadjiefthyvoulou et al., 2011b | Ecstacy | 12.48 | 3.27 | 29 | 16 | 1.68 | 9 |
| Montgomery et al., 2010 | Ecstacy | 80.4348 | 26.06 | 23 | 84.027 | 26.828 | 36 |
| Rendell et al., 2007 | Ecstacy | 0.478 | 0.1846 | 27 | 0.7 | 0.1954 | 34 |
| Weinborn et al., 2011 | Ecstacy | 7.23 | 0.96 | 31 | 7.29 | 1.07 | 16 |
| Zakzanis et al., 2003 | Ecstacy | 1.3 | 1 | 15 | 1.5 | 0.9 | 17 |
| Iudicello et al., 2011 | Methamphetamine | 6.67 | 1.31 | 39 | 7.42 | 0.95 | 26 |
| Rendell et al., 2009 | Methamphetamine | 0.575 | 0.157 | 20 | 0.833 | 0.162 | 20 |
| Behdrendt et al., 2015 | Tobacco | 0.17 | 0.65 | 10 | 0.25 | 0.63 | 12 |
| Heffernan et al., 2010b | Tobacco | 11.5 | 1.54 | 18 | 14.9 | 1.96 | 22 |
| Heffernan et al., 2012 Current | Tobacco | 8.88 | 2.2 | 27 | 12.1 | 0.81 | 12 |
| Heffernan et al., 2012 Previous | Tobacco | 11 | 1.32 | 18 | 12.1 | 0.81 | 12 |
| Heffernan et al., 2013b | Tobacco | 10.4 | 3.71 | 39 | 12.6 | 3.26 | 39 |
| Heffernan et al., 2014 | Tobacco | 10.5 | 2.87 | 24 | 14.3 | 1.94 | 24 |
| Jansari et al., 2013 | Tobacco | 94.44 | 13.71 | 19 | 94.44 | 13.71 | 19 |
| Marshall et al., 2016 | Tobacco | 13.85 | 2.52 | 20 | 16.48 | 2.48 | 13 |
| Terret et al., 2014 | Opiate | 0.76 | 0.31 | 26 | 0.95 | 0.1 | 30 |

***S6: Time based PM, means, standard deviations, and sample sizes for each study***

|  | |  | | Drug Group | | |  | Controls | |  |
| --- | --- | --- | --- | --- | --- | --- | --- | --- | --- | --- |
| Study | Drug | | Mean | | SD | n | Mean | | SD | n |
| Griffiths et al., 2012 | Alcohol | | 0.3 | | 0.25 | 23 | 0.41 | | 0.29 | 24 |
| Heffernan et al., 2012b | Alcohol | | 13.5 | | 2.33 | 28 | 15 | | 2.63 | 28 |
| Marshall et al., 2016 | Alcohol | | 14.67 | | 2.41 | 40 | 15.8 | | 2 | 13 |
| Platt et al., 2015 | Alcohol | | 0.59 | | 0.07 | 19 | 0.76 | | 0.06 | 18 |
| Weinborn et al., 2011 | Alcohol | | 7.14 | | 1.01 | 21 | 7.29 | | 0.9 | 16 |
| Gallagher et al., 2014 | Cannabis | | 61.07 | | 23.44 | 36 | 77.15 | | 22.05 | 32 |
| Hadjiefthyvoulou et al., 2011b | Cannabis | | 12.33 | | 5.65 | 12 | 15.11 | | 3.51 | 9 |
| McHale et al., 2008 | Cannabis | | -3.2 | | 1.89 | 20 | -1.5 | | 1.67 | 10 |
| Montgomery et al., 2012 | Cannabis | | 56.25 | | 26.75 | 20 | 75 | | 26.9 | 20 |
| Gallagher et al., 2014 | Ecstacy | | 47.37 | | 28.47 | 103 | 77.15 | | 22.05 | 32 |
| Hadjiefthyvoulou et al., 2011b | Ecstacy | | 10.45 | | 3.94 | 29 | 15.11 | | 3.51 | 9 |
| Montgomery et al., 2010 | Ecstacy | | 73.91 | | 24.4 | 23 | 67.36 | | 30.37 | 36 |
| Rendell et al., 2007 | Ecstacy | | 0.41 | | 0.16 | 27 | 0.66 | | 0.21 | 34 |
| Weinborn et al., 2011 | Ecstacy | | 6.39 | | 1.33 | 31 | 7.29 | | 0.9 | 16 |
| Iudicello et al., 2011 | Methamphetamine | | 5.51 | | 1.55 | 39 | 6.65 | | 1.36 | 26 |
| Rendell et al., 2009 | Methamphetamine | | 0.116 | | 0.15 | 20 | 0.416 | | 0.30 | 20 |
| Behdrendt et al., 2015 | Tobacco | | 55.39 | | 99.52 | 10 | 48.8 | | 44.15 | 12 |
| Heffernan et al., 2010b | Tobacco | | 10.7 | | 2.46 | 18 | 15.9 | | 2.47 | 22 |
| Heffernan et al., 2014 | Tobacco | | 7.66 | | 3.1 | 24 | 15 | | 2.35 | 24 |
| Jansari et al., 2013 | Tobacco | | 94.44 | | 13.71 | 19 | 88.89 | | 17.62 | 19 |
| Marshall et al., 2016 | Tobacco | | 12.3 | | 2.39 | 20 | 15.8 | | 2 | 13 |
| McHale et al., 2008 | Tobacco | | 1.5 | | 1.7 | 20 | 1.5 | | 1.67 | 10 |
| Terret et al., 2014 | Opiate | | 0.63 | | 0.29 | 26 | 0.84 | | 0.22 | 30 |
